# Optimal Geometric Parameters for 3D Electrodes in Bioelectrochemical Systems: A Systematic Approach

**DOI:** 10.1002/cssc.202001232

**Published:** 2020-08-14

**Authors:** Christopher Moß, Niklas Jarmatz, Janina Heinze, Stephan Scholl, Uwe Schröder

**Affiliations:** ^1^ Institute of Environmental and Sustainable Chemistry Technische Universität Braunschweig Hagenring 30 38106 Braunschweig Germany; ^2^ Institute for Chemical and Thermal Process Engineering Technische Universität Braunschweig Langer Kamp 7 38106 Braunschweig Germany

**Keywords:** microbial electrochemistry, microbial fuel cell, electrode design, electrochemical reactor, modular flow reactor

## Abstract

In this study, the performance of electroactive bacteria (EAB), cultivated inside tubular electrode ducts, is systematically investigated to derive predictions on the behavior of EAB under conditions limited by electrochemical losses. A modeling approach is applied to assess the influence of the electrochemical losses on the electrochemical performance and scaling characteristics of complex 3D structures, such as sponges and foams. A modular flow reactor is designed that provides laminar and reproducible flow conditions as a platform for the systematic electrochemical and bioelectrochemical characterization of 3D electrodes in bioelectrochemical systems (BES). The bioelectrochemical experiments are carried out in a set of reactors incorporating cylindrical electrodes exhibiting ducts of 1 cm length and different diameters ranging from 0.1 cm up to 1 cm. Single duct calculations are extrapolated to three dimensions through geometrical considerations; trends in 3D bioanode performance are demonstrated using the resulting simplified 3D structure. The combined experimental and modeling approach constitutes a framework for future studies on systematic electrode design.

## Graphical abstract

**Navigating the ducts**: The performance of mixed culture electroactive biofilms is studied under controlled conditions inside tubular electrodes with systematically altered duct geometries. Based on the experimental results, a modeling approach is applied to assess the influence of electrochemical losses on the performance and scaling characteristics of complex electrode structures.

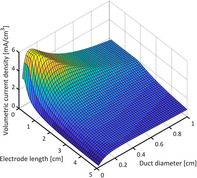

## Introduction

Microbial electrochemical technologies (METs) exploit biochemical catalysis by electroactive bacteria incorporated into an electrochemical cell.^1^, ^2^ MET applications include microbial fuel cells (MFCs)^3^ and microbial electrolysis cells (MECs),^4^ microbial electrosynthesis (MES),^5^, ^6^ bioremediation,^7^, ^8^ bioelectrochemical sensors^9^, ^10^ and microbial solar cells.^11^ In order to become economically competitive or feasible on larger scales, METs have to overcome bottlenecks caused by a relatively low conversion rate per electrode area (compared to conventional electrochemistry)^12^ in combination with a challenging upscaling of the reactor designs.^13^, ^14^, ^15^ Significant research efforts therefore aim towards improving the efficiency of underlying bioelectrochemical processes and developing efficient and cost‐effective electrode materials.^16^, ^17^, ^18^, ^19^, ^20^

In the context of microbial fuel cells and electrolysis cells, significant efforts have been put into the development of efficient bioanodes. The challenge is to design anodes that are mechanically stable, cost effective, highly conductive and come with good electrochemical properties, while at the same time providing a large surface area as a suitable EAB habitat.^13^, ^21^, ^22^, ^23^ Thus, several studies have been conducted to investigate the influence of electrode material,^24^, ^25^ surface structuring^26^, ^27^, ^28^ and chemical surface properties^29^, ^30^ on biofilm formation and bioanode performance.

In order to provide high specific and geometric surface area for bacterial colonization, a plethora of often complex and densely packed three‐dimensional electrode structures have been proposed. They include carbon cloth,^31^, ^32^ graphite felt,^33^, ^34^ fiber‐based electrode structures,^35^ carbon brushes,^36^, ^37^ granular electrodes,^38^, ^39^ layered structures,^40^, ^41^, ^42^ foams or sponges,^43^, ^44^, ^45^ and carbonated electrodes derived from natural^46^, ^47^, ^48^, ^49^ or artificial^50^, ^51^ precursors. A dense packing however can give rise to other limitations such as the clogging of the structure by biomass^43^, ^50^, ^52^ or electrochemical limitations owing to an unfavorable geometry and the typically low conductivity of solutions used in BES.^53^, ^54^ While bioanodes are usually designed with the possible bottlenecks in mind,^13^, ^23^, ^55^ the bottlenecks have not been quantified to an extent that would enable optimization of electrode structure towards a specific application. A systematic advancement in electrode development thus requires substituting the empiricism in the current electrode development by systematic and model‐based approaches, taking the often antipodal biological and physical requirements of bioelectrochemical processes into account.^28^, ^53^, ^54^, ^56^, ^57^, ^58^

In this context, a reproducible and controlled flow regime is a decisive prerequisite, since the flow conditions in a bioelectrochemical system may impact biofilm growth and performance.^59^, ^60^, ^61^ Such conditions are typically accomplished using continuous bioelectrochemical flow reactors, which may differ in design, size and complexity. Examples comprise micro‐scale reactors,^62^ two‐chamber MFCs,^63^ optically accessible flow reactors^64^ and pilot‐scale systems.^65^, ^66^, ^67^ Thereby, continuous operation also allows stationary cultivation and feeding conditions.^68^

In this study, a modular flow cell was designed containing graphite electrodes with a defined three‐dimensionality – adjusted via the length of the electrodes and via the diameter of bore channels (ducts) drilled through the electrodes. We used this setup to study the impact of the 3D electrode characteristics on the growth and performance of electroactive microbial biofilms. Thereby, an electrochemical and bioelectrochemical analysis in combination with model‐based calculations was applied aiming to derive predictions for macroscopic, multi‐channel structures, such as sponges and foams.

## Experimental Section

### Design of the electrochemical flow reactors

**Figure 1:**
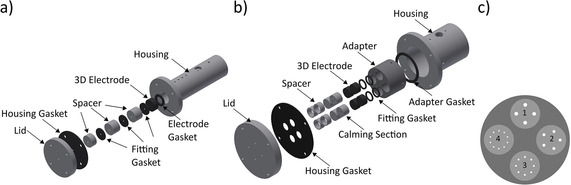
Explosion view of a) the single duct flow reactor, used for electrochemical characterization and b) the multiple duct flow reactor, used for the bioelectrochemical experiments. Flow direction from left to right. Four electrodes with a corresponding calming section of varying duct size and quantity as well as the spacers (1) to (4) can be inserted into the fourfold adapter of the multiple duct flow reactor c).

In order to systematically investigate the influence of the electrode geometry on the overall reactor performance, the experimental approach was divided into two steps resulting in the design and operation of two modular flow reactors – for the fundamental electrochemical characterization and for the bioelectrochemical experiments, respectively. Both reactors are based on a tubular geometry because this enables a simple accessibility and convertibility of the electrodes, the simultaneous application of multiple electrodes as well as a simplified scale‐up. Since the ohmic resistance of the liquid column inside the 3D electrodes was expected to play an important role for the overall reactor performance, the first experiments focused on the fundamental electrochemical characterization of the applied cylindrical 3D electrode design. For this purpose, a single duct flow reactor was designed to allow a fast and simple working electrode exchange. Thereby, the application of spacers allowed to use anodes of various lengths (Figure 1a). Owing to its single chamber, membrane‐free design, it is possible to easily test 3D electrodes of varying geometries in a simple three‐electrode setup while simultaneously ensuring a defined distance between the electrodes that are positioned in a row with respect to the flow direction. Accordingly, the calming section was sized in a way that a laminar flow regime can be assumed inside the duct of the working electrode for a Reynolds number of *Re*<100. Such reproducible flow conditions can be applied to link the flow characteristics of the reactor to the electrochemical electrode performance.

In a second step, cultivation experiments were performed to investigate the influence of the duct/electrode geometry on the performance of the bioanode. The developed multiple‐duct flow reactor expands the attributes of the single duct flow reactor, like the well‐defined laminar and reproducible flow conditions for *Re*<100, by the possibility to test multiple electrodes exhibiting e. g. varying duct diameters in one single reactor (Figure 1b). Four inlets allow the equal distribution of the cultivation medium over the entire profile of the working electrodes. Almost any given anode dimension can be implemented by the application of spacers and calming section attachments. It is also possible to vary the number of the working electrodes via electrode adapters (Figure 1c). In this study, the electrodes of each flow reactor contained tubular ducts of four different diameters to specifically allow the investigation of the influence of the electrode geometry on the bioanode performance.

The flow reactor housing as well as the spacers were made of Polyvinyl chloride (PVC‐CAW, W. Max Wirth GmbH, Germany). Flat gaskets (Butyl‐IIR‐plate – Shore 60°, 2 mm, RCT Reichelt Chemietechnik GmbH, Germany) with a diameter of 80 mm (single duct flow reactor) and 110 mm (multiple duct flow reactor) were used to seal the connection between the flow cell housing and the lid. Flat gaskets (silicone‐plate – Shore 55°, 2 mm, RCT Reichelt Chemietechnik GmbH, Germany) with a diameter of 25 mm were applied to seal the connection between the spacers and the working electrode. A rolling pin spring cotter (Rolling pin, N&H Technology GmbH, Germany) connected the graphite anodes. The graphite anodes were cut from polycrystalline graphite rods (CP Graphite, Germany) with a diameter of 25 mm and 20 mm for the single duct flow reactor and multiple duct flow reactor, respectively. For the experiments performed in the single duct flow reactor, one 4 mm duct was drilled into the working electrode. A reference electrode (Ag/AgCl, sat. KCl, Sensortechnik Meinsberg, Germany) was included in the reactor. Epoxy resin (R&G Faserverbundwerkstoffe GmbH, Germany) was used to connect the working electrode to the calming section and to insulate the cut surface of the electrode disk. A stainless‐steel wire (Linde Schweißtechnik GmbH, Germany) with a diameter of 1 mm was reeled to create a bundle, serving as the counter electrode.

**Figure 2:**
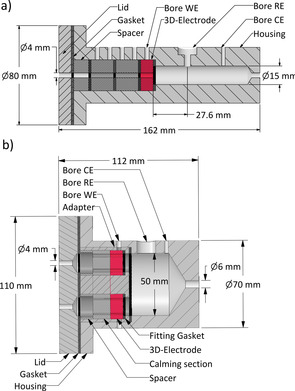
Sketch of the rip cut of the single duct flow reactor a) and the multiple duct flow reactor b). Flow direction from left to right. WE=working electrode, RE=reference electrode, CE=counter electrode.

The dimensions of the working electrodes were the main factor determining the reactor sizing. A minimal electrode length of 1 cm was chosen, as this size is similar to the electrode dimensions frequently used in BES electrode design studies.^69^ As visible in Figure 2, the positioning of the electrode in the single duct flow reactor creates a liquid column with a length of 27.6 mm and a diameter of 15 mm between the reference electrode and the closest possible position of the outlets of the anode duct. The anode ducts of the multiple duct flow reactor were prepared to feature diameters of 0.1 cm, 0.12 cm, 0.125 cm, 0.15 cm, 0.165 cm, 0.2 cm, 0.25 cm, 0.3 cm, 0.33 cm, 0.5 cm, 0.6 cm and 1 cm. We chose these duct diameters to span one order of magnitude to create a solid database for model calculations. Secondly, on the basis of our preceding study,^52^ in this size range a significant impact of biofilm growth or clogging was not to be expected. The number of ducts incorporated in the graphite electrodes was adjusted to obtain equal total surface areas for all 4 electrodes in each run (Table S1).

Dimensionless numbers help to characterize a system on a model scale and support a later upscaling. Hereby, Reynolds numbers characterize the fluid dynamic status and allow the discrimination between laminar and turbulent flow regimes. In order to ensure laminar flow conditions inside a tube or duct, an upstream calming section was integrated. This leads to a high reproducibility of the flow conditions.

The Reynolds number (*Re*) is defined in [Eq. (1)].

(1) $$Re=\frac{\rho \;d\;v}{\eta }$$

where *ρ* [kg m^−3^] is the density and *η* [kg s^−1^ m^−1^] the dynamic viscosity of electrolyte solution or cultivation medium, *d* [m] the hydraulic diameter of the duct and *v* [m s^−1^] the average fluid velocity inside the duct.^70^ The density and dynamic viscosity of the electrolyte solution/cultivation medium were approximated with the values for water at 35 °C.^71^ Here, Reynolds numbers between 1<*Re*<100 are of great interest, because for this range laminar flow conditions with a strong Reynolds number dependency occur. Thus, all reactors presented are sized for a maximum Reynolds number of *Re*_max_≈100.

Except for the experiments depicted in Figure S1, all abiotic electrochemical characterization experiments were performed without the application of a medium flow (*Re*=0). For the bioelectrochemical experiments, a constant Reynolds number of *Re*≈30 was set for all ducts by adjusting the diameter of the ducts while applying a constant volume flow by the pumps connected to the flow reactors. This *Re* value was chosen because for this range the experimental setup is well calculable, the biofilm inside the ducts is adequately supplied with nutrients and it is ensured that the ducts are not blocked by biofilm. At this point it needs to be kept in mind that e. g. waste water treatment plants – potential application sites for MFCs – generally provide flow conditions that correspond with very low Reynolds numbers (*Re*<1).^59^, ^72^ Here, a careful consideration of the transfer between the systems and the resulting scale‐up is inevitable.

To generate well‐defined laminar flow conditions for a duct with a diameter *d* [m], the minimum length of the calming section, *L*_c_ [m], is given by [Eq. (2)]:^70^

(2) $$L{}_{c}=0.06\;Re\;d$$

The calculated dimensions of the flow reactors are stated in Table S1. It is noteworthy that the required calming section for the electrode with a 1 cm duct diameter exceeds the provided calming section length of 14 mm.

The ohmic resistance of the liquid column inside the ducts can be determined applying Ohm's law. Thus, the correlation between the ohmic resistance *R* [Ω] and the length of the liquid column is linear and can be calculated by the equation for an electrical conductor [Eq. (3)]:

(3) $$R=\frac{\rho \;L}{S}$$

where *ρ* is the specific resistance [Ω mm^2^ m^−1^], *L* the length [m] and *S* [mm^2^] the cross section of the conductor.

### Electrochemical characterization

For the electrochemical characterization experiments, a test rig consisting of a peristaltic pump (ISMATEC® REGLO Digital MS‐4/12, Cole‐Parmer Instrument Company, LLC, USA) with butyl rubber pump tubes (Fluran HCA F‐5500‐A, 2.79 mm diameter Cole‐Parmer Instrument Company, LLC, USA) and a feed flask was connected to the single duct flow reactor (Figure 2 a) via butyl rubber tubes (ISMATEC Viton 4.8 1.6, Cole‐Parmer Instrument Company, LLC, USA) with a diameter of 4.8 mm. Butyl rubber was chosen for its low gas permeability, allowing the system to operate under anaerobic conditions. The flow cell was operated as three‐electrode setup using a VMP3‐potentiostat (Bio‐Logic Science Instruments SAS, France. A conductivity meter (Cond 315i/SET, WTW Wissenschaftlich‐technische‐Werkstätten GmbH, Germany) was used to measure the conductivity of a serial dilution of sulfuric acid (96 %, Carl Roth GmbH & Co. KG, Germany) as well as of the cultivation medium and of a wastewater sample from the local wastewater treatment plant (Steinhof, Braunschweig). Potassium hexacyanoferrate(II)trihydrate (Carl Roth GmbH & Co. KG, Germany) served as a Fe^2+^/Fe^3+^ redox system for the electrochemical characterization of the system. In preparation of the experiment, the graphite electrodes were sonicated in 0.2 M sulfuric acid for at least 5 min. After the flow cell reactor was assembled, the test rig was flushed with 0.2 M sulfuric acid. The respective solution was preheated to 35 °C and purged with gaseous nitrogen for at least 5 min while being circulated by the pump.

**Figure 3:**
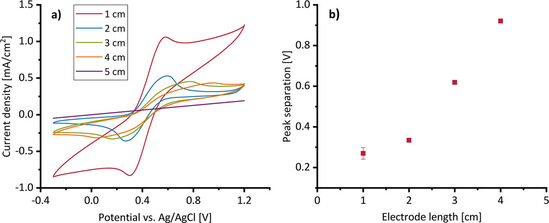
a) Cyclic voltammograms a 20 mM solution of K_4_[Fe(CN)_6_] in 200 mM sulfuric acid, recorded in the single‐duct cell, as a function of the working electrode (duct) length; b) resulting peak‐to‐peak separation. The scan rate was 5 mV s^−1^.

Cyclic voltammetry (CV) was carried out at varying potential ranges, scan rates and concentrations of electrolyte and redox species (if applied). To investigate the influence of the applied Reynolds number (Figure S1), the concentrations of sulfuric acid (electrolyte) and potassium hexacyanoferrate(II) (redox species) was set to 100 mM and 1 mM, respectively. The length of the electrode and the duct diameter were amounted to 1 cm and 0.4 cm, respectively (Figure 2 a). For the investigation of varying electrode geometries (Figure 3), the sulfuric acid concentration was adjusted to 200 mM and the hexacyanoferrate(II) concentration was set to 20 mM. The electrode length was adjusted between 1 cm and 4 cm. If not stated otherwise, a scan rate of 5 mV s^−1^ was applied. For all measurements, three CV cycles were recorded while the second cycle was used for the data evaluation.

Electrochemical impedance spectroscopy (EIS) was performed for the single duct reactor (Figure 2 a) with a working electrode potential of 0.2 V vs. Ag/AgCl reference electrode and a sinus amplitude of 20 mV. EIS was performed over a frequency range of 100 kHz to 1 Hz to determine the ohmic resistance of the reactor at varying electrolyte concentrations. The electrolyte (sulfuric acid) concentration was adjusted from 0.625 mM to 200 mM. A 20 mm spacer was placed between the working electrode and the reference electrode to increase the length of the liquid column. The experimental results were compared to values calculated based on the expected conductivity of the liquid column. For the experiments regarding a varying duct length, EIS was performed over a frequency range of 1 MHz to 100 Hz and the electrolyte concentration (sulfuric acid) was adjusted to 200 mM. The length of the electrode and the duct diameter were adjusted from 1 cm up to 5 cm for the single duct flow reactor (Figure 2 a).

### Bioelectrochemical measurements

Peristaltic pumps (ISMATEC® MCP Standard – CA8, Cole‐Parmer Instrument Company, LLC, USA) were used for the cultivation experiments with the multiple‐duct flow reactor. In order to assure an equal electrolyte/medium distribution between the four electrodes and to allow the discharge of gas bubbles, the reactors were mounted vertically. To minimize statistic deviation, the three replicates of the cultivation experiments were performed simultaneously in three independent runs using three identical test rigs (see table S1). The test rigs were set up using butyl rubber pump tubes (FC (F‐4040‐A), 2.79 mm diameter, Cole‐Parmer Instrument Company, LLC, USA) in combination with butyl rubber tubes exhibiting a diameter of 2.5 mm (Fluidvit FPM‐tubes, 2.5 mm diameter, Pro Liquid GmbH) and 4.8 mm (ISMATEC Viton 4.8 1.6, Cole‐Parmer Instrument Company, LLC, USA) to connect the experimental rig components. Every test rig was controlled by one potentiostat (MPG2, Bio‐Logic Science Instruments SAS, France). A data logger (Agilent 34970 A, Agilent Technologies, Inc., USA) recorded the current of the four working electrodes simultaneously. For biofilm cultivation and operation, the potential was set to 0.2 V vs. Ag/AgCl. The medium flasks that were connected to the flow reactors were purged with gaseous nitrogen for at least 15 min prior to the start of the experiment and further three times per week in order to remove dissolved oxygen from the culture medium. The medium was a modified standard cultivation medium,^73^ using 10 mM acetate as sole carbon source. The microbial inoculum consisted of a secondary culture containing mostly *Geobacter anodireducens* enriched from the primary wastewater of the local wastewater treatment plant (Steinhof, Braunschweig).^51^ The bioelectrochemical setups were operated inside an incubation hood (RCS, LTF Labortechnik, Germany), at a constant temperature of 35 °C. A volume flow of 2 mL min^−1^ (Re≈6) was kept constant until the bacterial culture transitioned from the lag phase into the exponential growths phase. The volume flow was then increased to 10.26 mL min^−1^ (run 1 and 2) as well as 6.14 mL min^−1^ (run 3) for the remaining runtime to ensure a Reynolds number of *Re*≈30. The experiments were terminated after four batch cycles (complete exchange of cultivation medium after a decrease of measured current of at least half of the maximum current). The total experiment duration was thereby approximately one month.

## Results and Discussion

### Electrochemical characterization

Prior to the conduction of bioelectrochemical experiments, the single‐duct flow reactor was analyzed towards its electrochemical characteristics and towards potential electrochemical limitations. In order to confirm that the reactors provide stable and laminar flow conditions, the volume flow (and thus the Reynolds numbers) was increased stepwise while recording cyclic voltammograms of a hexacyanoferrate redox system (see Figure S1). The data show that the current response of the system is stable for Reynolds numbers below *Re*_max_=100. Interestingly, the curves corresponding to *Re*=420 as well as *Re*=560 exhibit a trembling in the steady state current region. It can be assumed that this is owing to an exceeding of the maximum Reynolds number, which was considered for the reactor design.

Further voltammetric experiments were performed comprising a variation of the electrolyte concentration with and without addition of hexacyanoferrate, a variation of the hexacyanoferrate concentration and a variation of scan rate (Figure S2–S5). The data imply that the flow reactors are capable of supplying laminar flow conditions. There are no severe electrochemical limitations with respect to the reactor design. The voltammetric behavior was further investigated with respect to the length the electrode (and thus of the duct). As expected, an increasing length leads to a significant increase in the peak‐to‐peak separation, which above 4 cm length even exceeds the potential window of the voltammogram (Figure 3). The peak currents decrease rapidly, while the peak shape becomes broader and less sharp. The increasing peak separation can be explained by an ohmic overpotential that increases steadily with the growing liquid column inside the electrode duct – from the opening of the duct (at the electrode surface) into its depth. Since every segment of the duct is exposed to a different potential, the electrode shows a mixed potential, explaining the peak broadening. These results indicate that a significant potential drop can occur even for a relatively short duct length, emphasizing the role of the ohmic overpotential as a main factor affecting the performance of a 3D electrode.

The experiments conducted for the single duct flow reactor allow the quantification of the ohmic resistance for a working electrode positioned at a varying distance to the reference electrode (Figure S6). The data show a linear increase of the ohmic resistance with the liquid column in the duct. The resistance values obtained for a duct length of 0 cm result from the liquid column between the closest possible position of the working electrode and the reference electrode (≈30 Ω).

### Bioelectrochemical results

**Figure 4:**
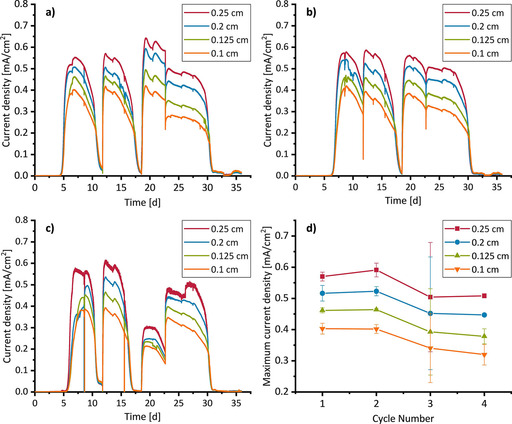
Chronoamperometry curves a), b), c) and maximum current densities d) of run no. 1 including the following duct diameters: 0.1 cm, 0.125 cm, 0.2 cm and 0.25 cm.

The results of the bioelectrochemical experiments, which were performed in three independent replicates, are displayed in Figures 4, S7 and S8. Each reactor contained four electrodes with different duct diameters (but comparable total surface areas, Table S1) resulting in three sets of chronoamperograms (CAs) (Figure 4a–c). From these CAs, the maximum current densities of the feeding cycles can be extracted as triplicates (Figure 4d).

**Figure 5:**
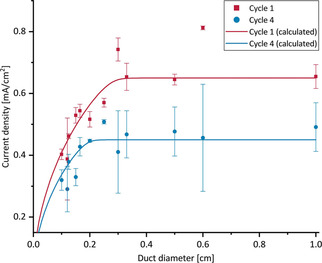
Influence of the duct diameter on the maximum current density of the bioanode (length 1 cm): experimental data (Figures 4, S7, S8) and bioelectrochemical calculations (Figures S7 and S8); the maximum current densities used for the calculations (0.65 mA cm^−2^and 0.45 mA cm^−2^) are chosen to reflect the experimental results.

In total, the current density data were determined for 12 different diameters, ranging from 0.1 cm to 1 cm. Beside a gradually decreasing electrode performance over time, the CAs show a strong dependence of the current densities on the channel diameters (see Figures S7 and S8). To elucidate this behaviour in detail, Figure 5 plots the maximum current densities against the duct diameters.

Figure 5 illustrates that, whereas the current density at channel diameters above 0.2 cm are independent on the channel diameter, diameters below 0.2 cm lead to strongly decreasing current densities. Based on the electrochemical characterization described above (Figures 3 and S6), the decreasing current density at smaller channel diameters can be ascribed to the increasing ohmic resistance inside the ducts and the resulting potential shift. Therefore, the constant current measured for larger diameters can be assumed to represent unhindered conditions for the EAB.

**Figure 6:**
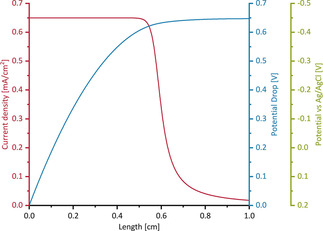
Potential (blue line) and current (red line) distribution in a duct of 0.1 cm diameter over its length. The green y‐axis illustrates the potential relative to the Ag/AgCl reference electrode.

To validate this assumption and to quantify the electrocatalytic behavior of the EAB for different geometries, the potential and current distribution along the ducts were calculated using Ohms law and the Nernst‐Monod‐equation,^74^ thereby applying the measured maximum current densities for unhindered conditions (i. e. 0.65 mA cm^−2^ for cycle 1 and 0.45 mA cm^−2^ for cycle 4) and a potential of −0.155 V vs. SHE^75^ as the literature value for the half‐saturation potential of the EAB catalytic activity as framework. Using the Nernst‐Monod‐Equation, the expected performance of the EAB can be derived for any location inside the structure as long as the EAB performance (i. e. current density) under unhindered conditions and the potential shift occurring at that point are known. An example of the resulting potential and current density distribution is shown in Figure 6 for a duct with a diameter of 0.1 cm. The current densities calculated for each diameter are represented by the line graphs in Figure 5. It can be seen that they describe the experimental data reasonably well.

The dominating role of the ohmic resistance confirms the data of the abiotic electrochemical experiments described above (Figures 3 and S6), and is also in agreement to previous BES studies.^58^, ^76^, ^77^

**Figure 7:**
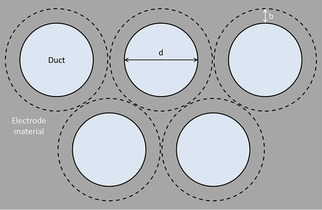
Sketch illustrating the extrapolation of single duct data to volumetric values; d: duct diameter, b: wall thickness representing the amount of material needed for structural integrity/conductivity.

Naturally, technical 3D electrodes do not contain a single channel or pore, but multiple of these structural elements. To approximate the behavior of such a 3D bioanode, the results from single duct calculations have to be extrapolated to three dimensions. For this purpose, we use a model based on a tight packing of parallel cylindrical tubes (Figure 7). It bears a noticeable resemblance to electrode designs featuring channels or sponge like structures frequently proposed in literature.^40^, ^41^, ^43^, ^44^, ^77^ It can thus be used as a simplified model to represent three dimensional electrode geometries.

Hereby *d* [cm] in Figure 7 describes the duct diameter and *b* [cm] is the wall thickness (equivalent to a tube wall thickness) representing the thickness of the electrode material. Thus, the surface area per unit volume can be calculated as follows [Eq. (4)]:

(4) $$\frac{A}{V}=\delta \cdot \left(1-\frac{4db+4b^{2}}{d^{2}+4db+4b^{2}}\right)\cdot \frac{4}{d}$$

**Figure 8:**
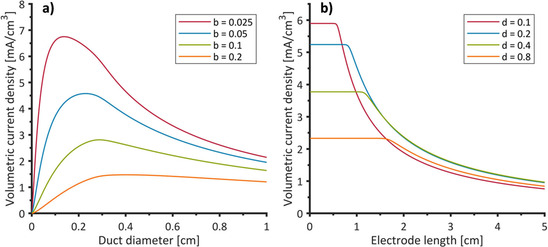
a) Volumetric current density of an electrode of 1 cm length plotted against duct diameter for different values of the wall thickness b (Figure 7); b) Volumetric current density plotted against electrode length for different duct diameters (b=0.05 cm).

Hereby, *A* [cm^2^] is the inside area of the ducts (Figure 7) and represents the (geometric) surface area of the electrode, V [cm^3^] is the electrode volume and δ constitutes the packing efficiency for the densest two‐dimensional packing of circles (δ =0.907 for the hexagonal packing arrangement). As shown in Figure S10, the surface area per volume of a resulting 3D electrode mainly depends on the absolute values of *d* and *b* as well as on their ratio. In conjunction with the above described bioelectrochemical data and calculations, the resulting volumetric current density of a 3D bioanode can be calculated. Figure 8 illustrates the dependence of volumetric current density on geometrical parameters for two different cases: a) volumetric current density plotted against duct diameter for an electrode of 1 cm length with *b* as variable and b) volumetric current density plotted against electrode length with *d* as the variable (and *b*=0.05 cm).

The volumetric current density increases towards lower values of *b* because the ducts can be packed more tightly into a given volume (Figure 8 a). However, the thickness of the material can be decreased only to a certain point before the conductivity and the mechanical integrity of the electrode material become limiting.^13^, ^57^ The potential drop increases along the duct length (Figure 6) leading to conditions where the EAB will eventually become inactive. In this case, the volumetric current density of the bioanode decreases with increasing electrode length. This trend becomes more pronounced for smaller duct diameters owing to their higher solution resistance. This circumstance can result in structures with wider ducts achieving higher volumetric current densities despite providing significantly less surface area per volume (Figure 8 b).

**Figure 9:**
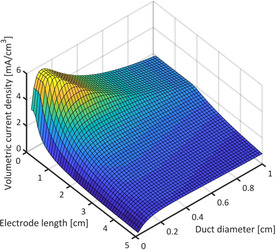
3D plot of the volumetric current density drawn against electrode length (up to 5 cm) and duct diameter (up to 1 cm) for b=0.05 cm.

It can be summarized, that the volumetric current density increases for decreasing duct diameters, yet, with the increase being limited by the ohmic resistance of the solution – depending on the electrode length. Figure 9 assembles the different effects into a 3D plot of the volumetric current density against the duct diameter and the electrode length, thus summarizing the trends illustrated in Figure 8.

However, while the volumetric current density decreases with increasing duct length and duct diameter, the total current produced by the bioanode still increases towards higher volumes when scaled uniformly in all space directions (Figure S11). While uniform scaling does increase the total current, the volumetric current density will decrease with electrode size. Retaining high current densities can only be achieved by adding more ducts and thus scaling only the lateral dimension of the electrode. This means that depending on the current density that is generated per unit area, the volumetric bioanode performance might depend more strongly on electrochemical limitations than on the specific surface area of the electrode structure.

Instead of considering duct length and diameter separately, a geometric parameter *G* can be introduced as the ratio of the duct length *L* [cm] and the cross‐sectional area *S [cm^2^]* of the duct [Eq. (5)]:

(5) $$G=\frac{L_{\mathrm{duct}}}{S_{\mathrm{duct}}}$$

**Figure 10:**
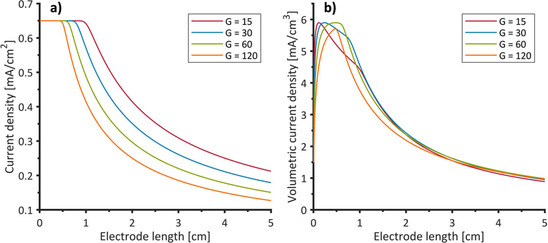
Current density a) and volumetric current density b) plotted against electrode length for electrodes with different duct geometries (ratio of duct length and duct base area, denoted as G [cm^−1^] for b=0.05 cm.

Thus, for a distinct *G* value, the total ohmic resistance of the duct is the same independent of the individual duct size. Figure 10 shows calculated bioanode performances for several geometries based on the experimental data: a) current density plotted against the electrode length and b) volumetric current density plotted against the electrode length for different values of G. The onset of surface‐related current density decrease is shifted towards smaller electrode lengths for higher values of G (Figure 10a). The volumetric current density depends on the electrodes’ available surface area as long as the bioanodes performance is not limited by the ohmic resistance (Figure 10b).

The maximum of each curve denotes the electrode length corresponding to the highest surface area for a given duct geometry. The volumetric current density then decreases owing to a reduction of surface area and subsequently (Figure 10b, red line and blue line) or directly (Figure 10 b, green line, orange line) owing to the ohmic resistance. It can also be seen in Figure 10b that for *G*=120 the curve does not reach the maximum possible current density. This is because the current density falls off owing to ohmic resistance before the maximum surface area for this geometry is reached. Figure 10 illustrates that by calculating the expected volumetric current densities based on bioelectrochemical data, an optimal duct geometry (i. e. electrode structure) can be derived with respect to a specific duct length. Thereby, a significant increase in volumetric current density can be achieved compared to suboptimal electrode structures.

**Figure 11:**
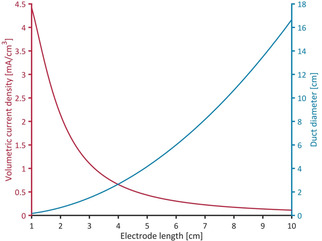
Duct diameter (blue line) calculated to reach 80 % of the maximum current density per area and corresponding volumetric current density (red line) plotted against the electrode length for b=0.05 cm.

To provide a suitable habitat for EAB over the integral electrode surface area, the appropriate duct diameter can be calculated based on the solution conductivity and experimental data or expected current density values (Figure 11). Figure 11 shows the calculated diameters and the resulting volumetric current density plotted against the electrode length.

**Figure 12:**
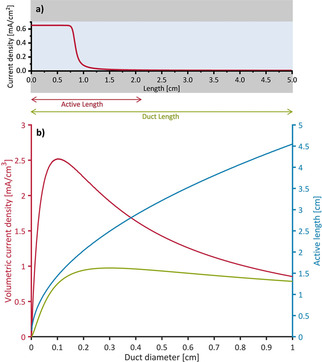
a) Current density distribution for a duct of 0.2 cm diameter illustrating active and inactive fractions of the duct; b) Length of the active part (current density >1 % of maximum current density) of the ducts (blue line) and volumetric current density with regard to the active part of the ducts (red line) and with regard to the full duct length (green line) calculated for an electrode length of 5 cm plotted against the duct diameter.

Calculations were based on the experimental data with the requirement of the current density per area equating to 80 % of the expected maximum values (owing to expected variance in EAB performance (Figures 4, 5, S7, S8)). It can be seen that the diameter that is necessary to provide 80 % of maximum current density per area increases drastically with increasing electrode length. As such, the electrodes’ available surface area and, as a result, the volumetric current density decrease accordingly. In a similar approach, the active duct length representing the maximum electrode dimensions that can support EAB catalytic activity is calculated. This is illustrated in Figure 12 a for a duct of 5 cm length and 0.2 cm diameter: the red arrow denotes the active fraction of the length and the green arrow indicates the full length of the duct. For calculations, the active duct length has been defined as the length of a duct up to the point where the current is >1 % of the maximum current produced under unhindered conditions (Figure 12a, red arrow). Figure 12b shows the active length (blue line) and the corresponding volumetric current densities with respect to the active length (red line) as well as to the full length of 5 cm (green line). It can be seen that the active length increases towards higher duct diameters, almost approaching the electrode length at a diameter of 1 cm. The volumetric current density, with respect to the active length (red line), is highest for smaller diameters exhibiting a distinct maximum around 0.1 cm. For the volumetric current density with respect to the full electrode length, the maximum shifts towards a duct diameter around 0.3 cm and decreases by a factor of approximately 2.5. Also, larger diameters produce similar current densities in this case as the loss of area per volume is compensated by the gain of active electrode length. As the duct diameter approaches 1 cm, the red and green lines converge as the active length approaches the total length of the electrode.

The calculations show that a significant increase in volumetric current density can be achieved by tailoring the electrode dimensions towards the active length or vice versa tailoring the electrode structure to the required electrode size. While performance losses owing to solution resistance cannot be completely circumvented by means of electrode design, a systematic approach on electrode design can be applied to minimize such losses.

## Conclusions

In this study, the successful development of a modular flow reactor design as an adaptable platform for studying 3D electrodes in bioelectrochemical systems was presented. Favorable laminar flow conditions inside the electrode can be supplied up to a maximum Reynolds number of *Re*_max_=100. The modular design of the simplified single duct flow reactor proved to be highly suitable for electrochemical parameter screening. The more complex multiple duct flow reactor allowed the evaluation of 3D electrode designs with respect to the performance as a bioanode during biofilm cultivation experiments. The scalable, modular reactor design can be applied for studies under a varying reactor complexity – depending on the experimental demand.

Mixed culture EAB were cultivated inside the multiple duct flow cell under reproducible conditions on tubular graphite electrodes with systematically varied duct diameters to assess the effect of the electrode geometry on EAB performance. For smaller diameters, an increase in current density was observed that leveled off towards a plateau of constant current density for larger diameters. The experimental data were approximated to good agreement by calculating the potential and current distribution inside the ducts using the experimental values obtained for higher duct diameters. The assumption was verified that the current density decrease towards small diameters was owing to the ohmic resistance and it was possible to model the experimental data based on Ohms law and the Nernst‐Monod equation.

Geometric considerations were applied to extrapolate the results from single duct calculations to a simplified 3D electrode geometry, which was used to calculate volumetric current densities and derive trends based on duct diameter/geometry, material thickness, and electrode dimensions. The results show that a tightly packed structure is only advantageous for flat electrode designs (i. e. short duct length), high ionic conductivities and/or very low biofilm performance. A low material thickness is assumed to support the performance of the system, but the value has to be carefully adjusted based on material conductivity and mechanical stability.

A systematic approach to 3D bioanode designs proves advantageous compared to purely empiric experiments, as the results are transferable and scalable. A modeling approach based on experimental data has been identified as a promising way towards systematic electrode development. It can be used to propose design and scaling strategies for a high volumetric performance or turnover rate.

Further studies should validate the results of this study for more complex substrate conditions. They may also expand the above considerations to incorporate potential limitations that occur across electrochemically active biofilms, such as mass transfer limitations and ohmic resistances and take into account the effects of biofilm growth inside the 3D structures.

## Conflict of interest

The authors declare no conflict of interest.

## Supporting information

As a service to our authors and readers, this journal provides supporting information supplied by the authors. Such materials are peer reviewed and may be re‐organized for online delivery, but are not copy‐edited or typeset. Technical support issues arising from supporting information (other than missing files) should be addressed to the authors.

Supplementary
