# Supplementary material for: Optimal Geometric Parameters for 3D Electrodes in Bioelectrochemical Systems: A Systematic Approach
Source: ChemSusChem. 2020 Aug 14;13(18):5119–29. doi: 10.1002/cssc.202001232 (PMC7540030; doi:10.1002/cssc.202001232)
Supplement: Supplementary file 1 — Supplementary [file CSSC-13-5119-s001.pdf]

ChemSusChem

Supporting Information

Optimal Geometric Parameters for 3D Electrodes in Bioelectrochemical Systems: A Systematic Approach

Christopher Moß[+], Niklas Jarmatz[+], Janina Heinze, Stephan Scholl, and Uwe Schröder*© 2020 The Authors. Published by Wiley-VCH GmbH. This is an open access article under the terms of the Creative Commons Attribution License, which permits use, distribution and reproduction in any medium, provided the original work is properly cited.This publication is part of a Special Collection highlighting "The Latest Research from our Board Members". Please visit the Special Collection at https://bit.ly/cscBoardMembers

Table S1: Dimensions and parameters of the examined 3D anodes for the cultivation experiments in the multiple duct flow reactor. For the calculations a constant Reynolds number of Re = 30 was set.

Duct diameter [mm]	Length calming section [mm]	Mean duct velocity [m s^{-1}]	Duct quantity	Electrode volume flow [mL min^{-1}]	Electrode surface area [mm^2]
**First run**					
1	1.8	0.022	10	10.23	314
1.25	2.25	0.017	8	10.23	314
2	3.6	0.011	5	10.23	314
2.5	4.5	0.009	4	10.23	314
1	1.8	0.022	10	10.23	314
**Second run**					
1.65	2.97	0.013	6	10.23	314
3.3	5.94	0.007	3	10.23	314
5	9	0.004	2	10.23	314
10	18	0.002	1	10.23	314
**Third run**					
1.2	2.16	0.018	5	6.14	188
1.5	2.7	0.014	4	6.14	188
3	5.4	0.007	2	6.14	188
6	10.8	0.004	1	6.14	188

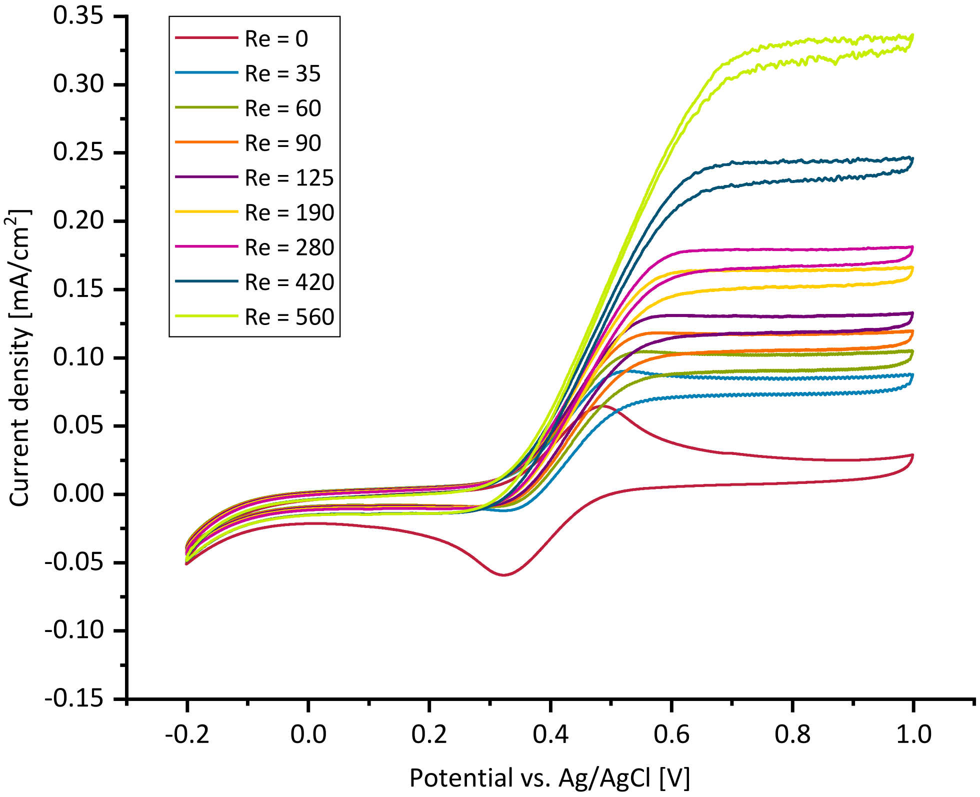

Figure S1: Cyclic voltammetry of a 1 mM solution of $K_4[Fe(CN)_6]$ in 100mM sulfuric acid at a varying Reynolds numbers. The scan rate was 5 mV s^{-1}.

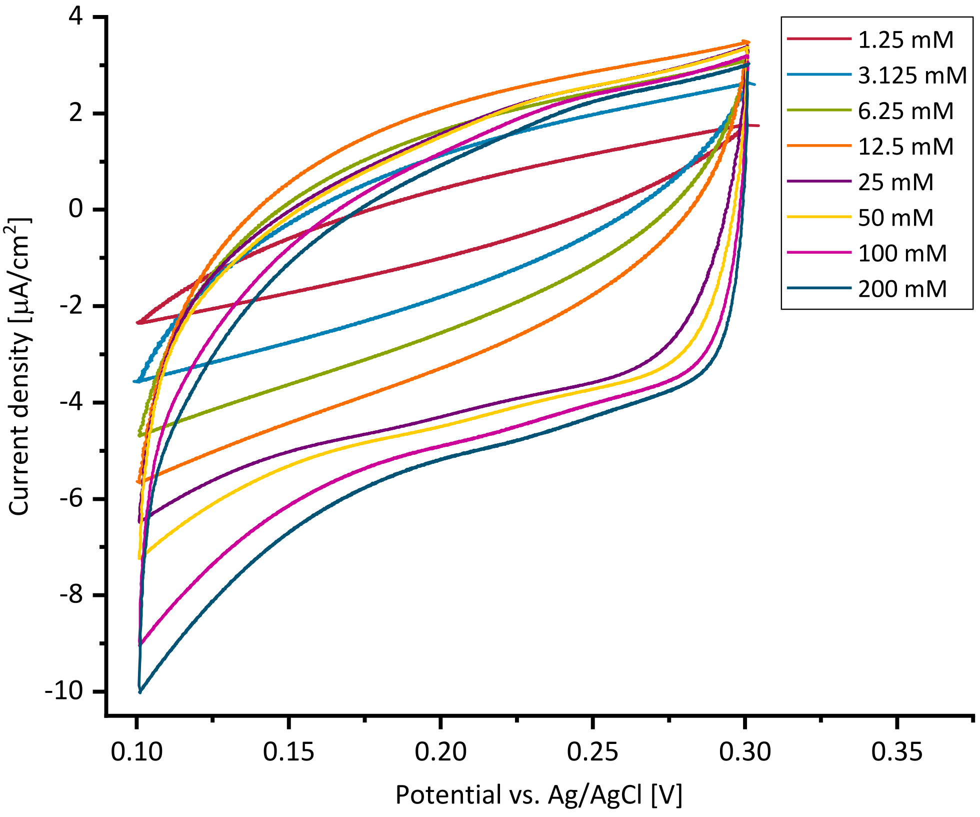

Figure S2: Cyclic voltammetry measurements comparing the effect of a varying electrolyte (sulfuric acid) concentration. The scan rate is set to 1 mV s^{-1}. The length of the electrode and the duct diameter were amounted to 10 mm and 4 mm, respectively.

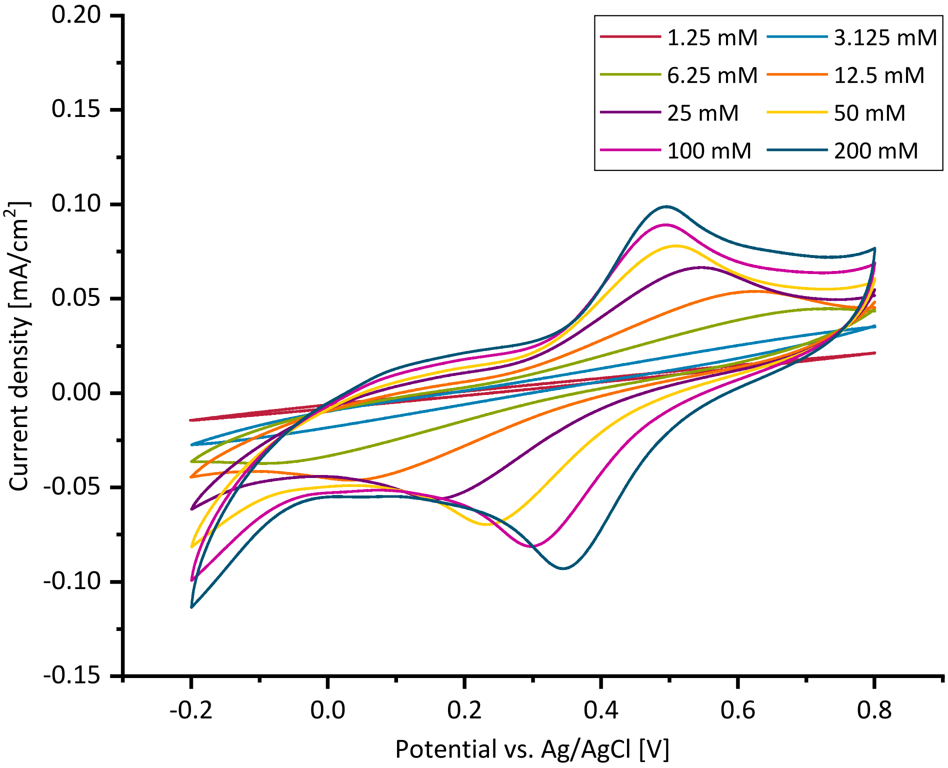

Figure S3: Cyclic voltammetry measurements comparing the effect of a varying electrolyte (sulfuric acid) concentration with a constant analyte (potassium hexacyanoferrate(II) trihydrate) concentration of 1 mM. The scan rate is set to 5 mV s^{-1}. The length of the electrode and the duct diameter were amounted to 10 mm and 4 mm, respectively.

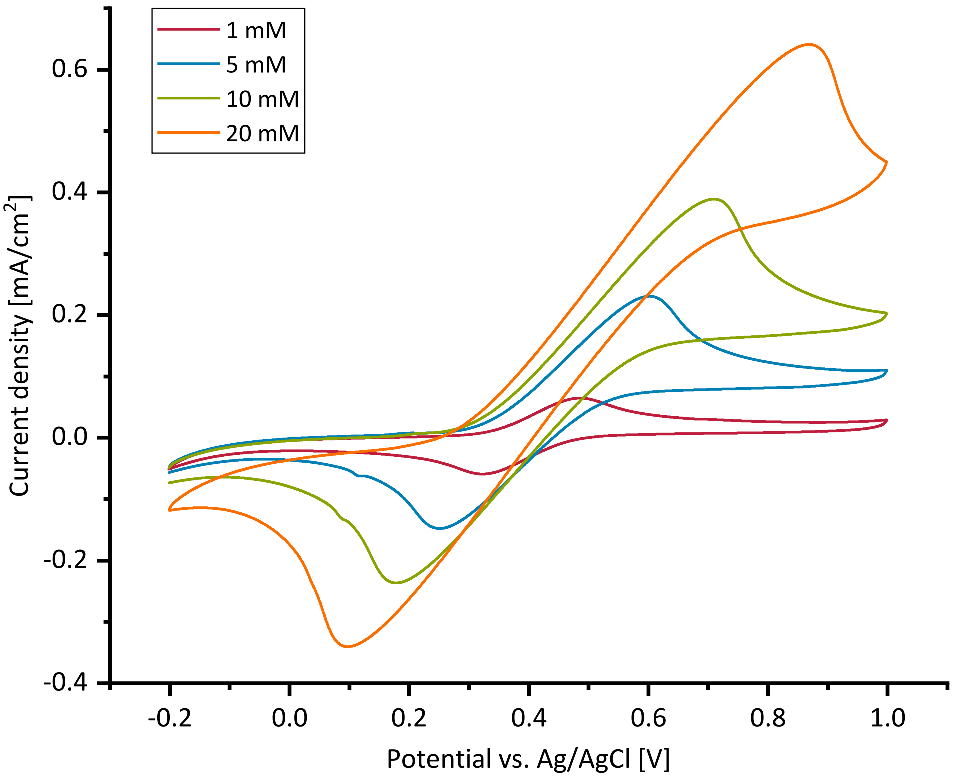

Figure S4: Cyclic voltammetry measurements comparing the effect of a varying analyte (potassium hexacyanoferrate(II)trihydrate) concentration. The electrolyte (sulfuric acid) concentration was set to 100 mM. The scan rate is set to 5 mV s^{-1}. The length of the electrode and the duct diameter were amounted to 10 mm and 4 mm, respectively.

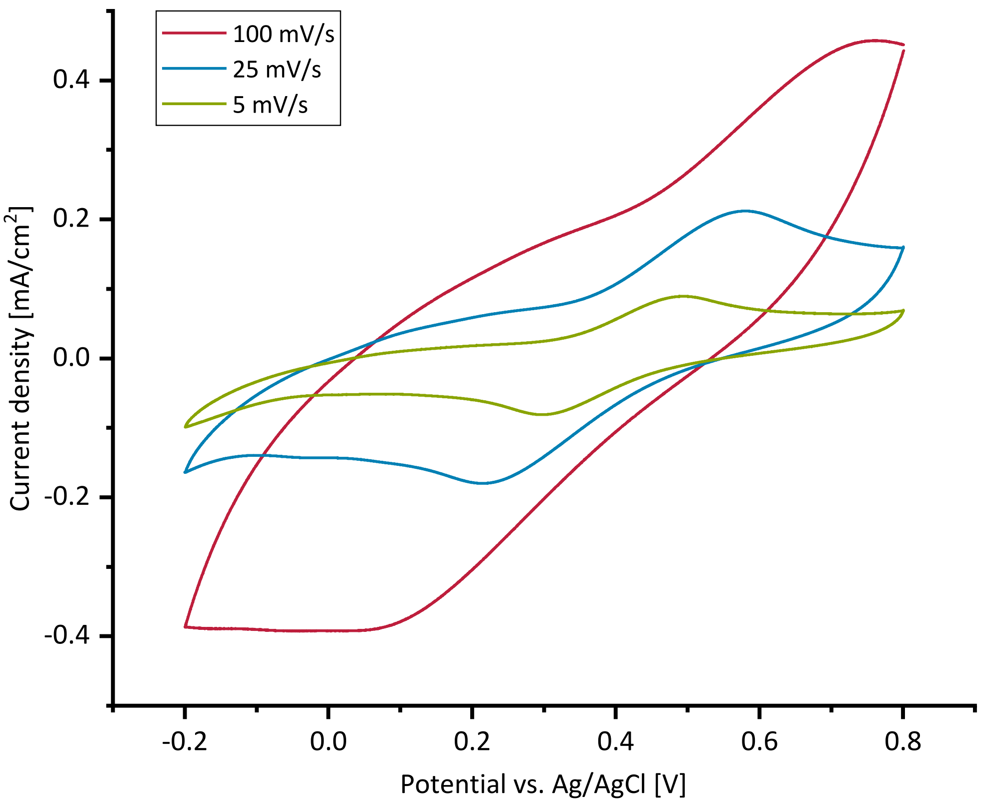

Figure S5: Cyclic voltammetry measurements comparing the effect of a varying scan rate. The electrolyte (sulfuric acid) and analyte (potassium hexacyanoferrate(II)trihydrate) concentration was set to 100 mM and 1 mM, respectively. The length of the electrode and the duct diameter were amounted to 10 mm and 4 mm, respectively.

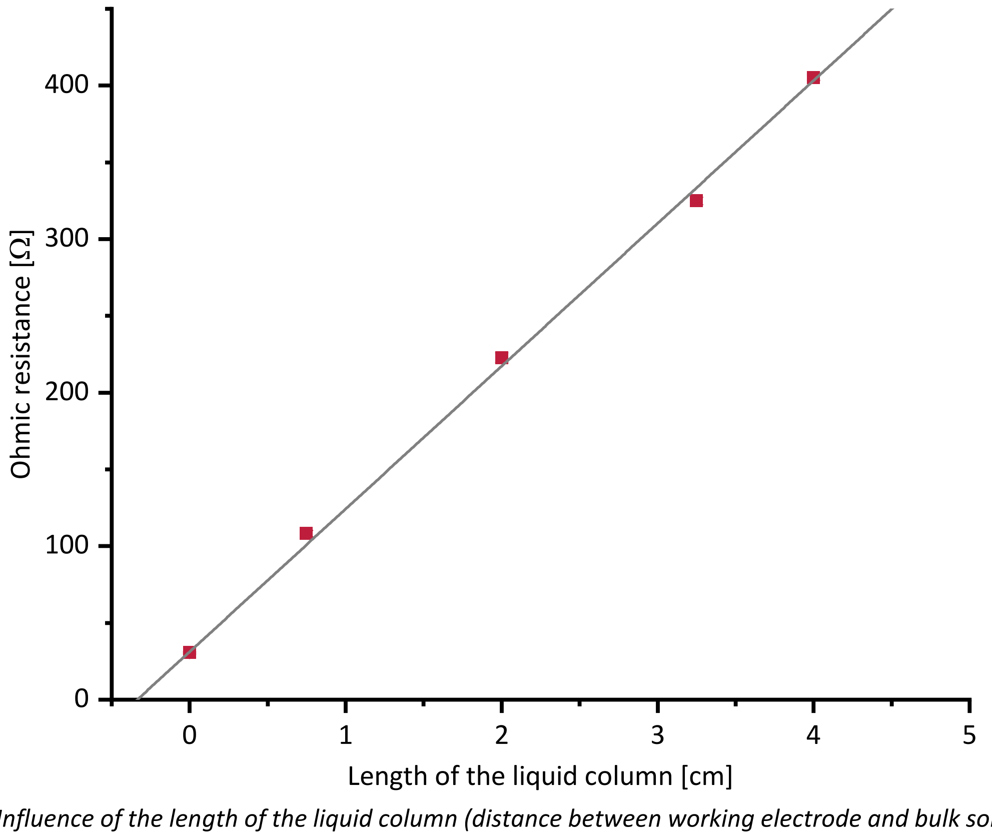

Figure S6: Influence of the length of the liquid column (distance between working electrode and bulk solution) on the ohmic resistance of the liquid column determined via electrochemical impedance spectroscopy.

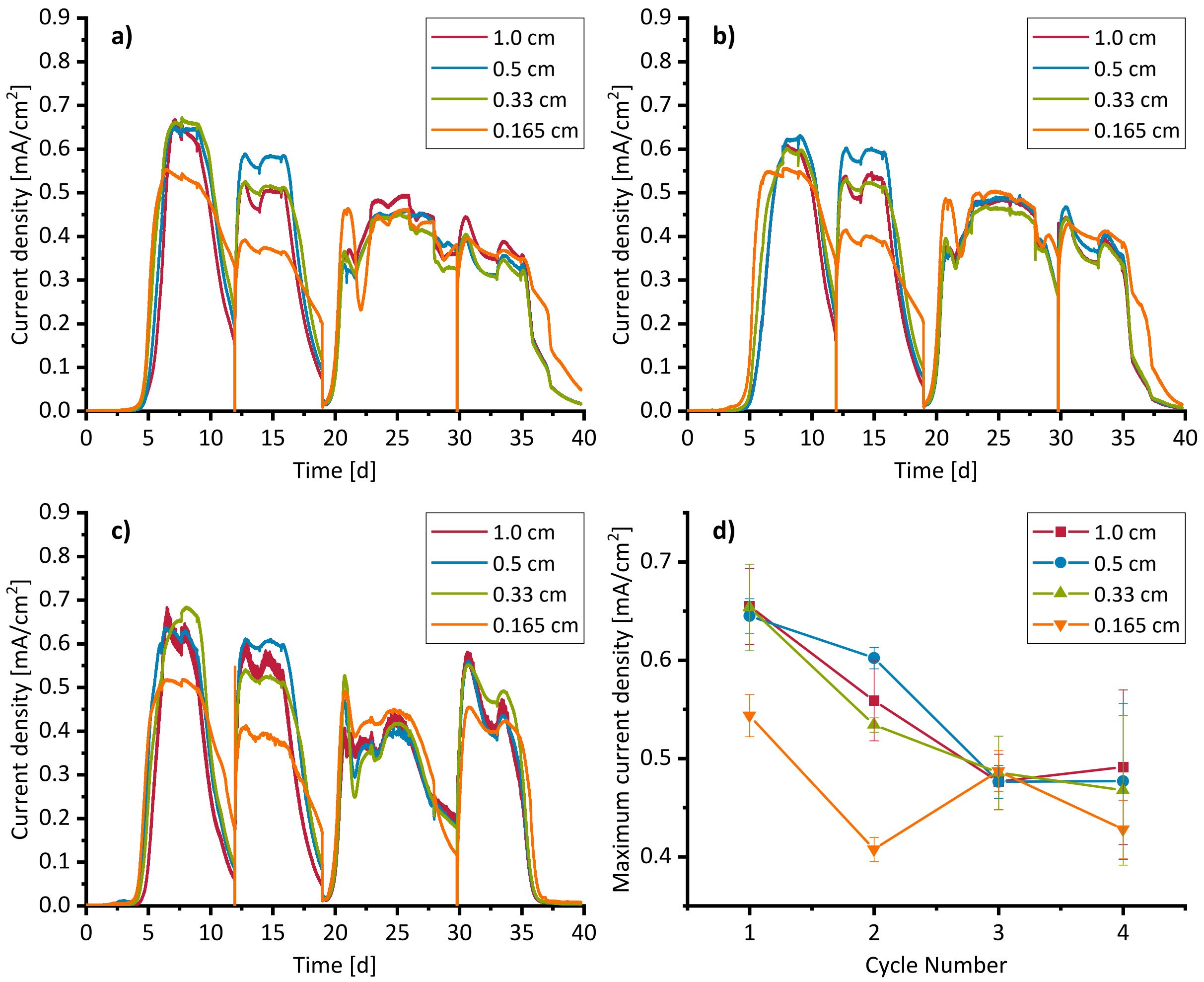

Figure S7: Chronoamperometry curves a), b), c) and maximum current densities d) of run no. 2 including the following duct diameters: 0.165 cm, 0.33 cm, 0.5 cm and 1.0 cm

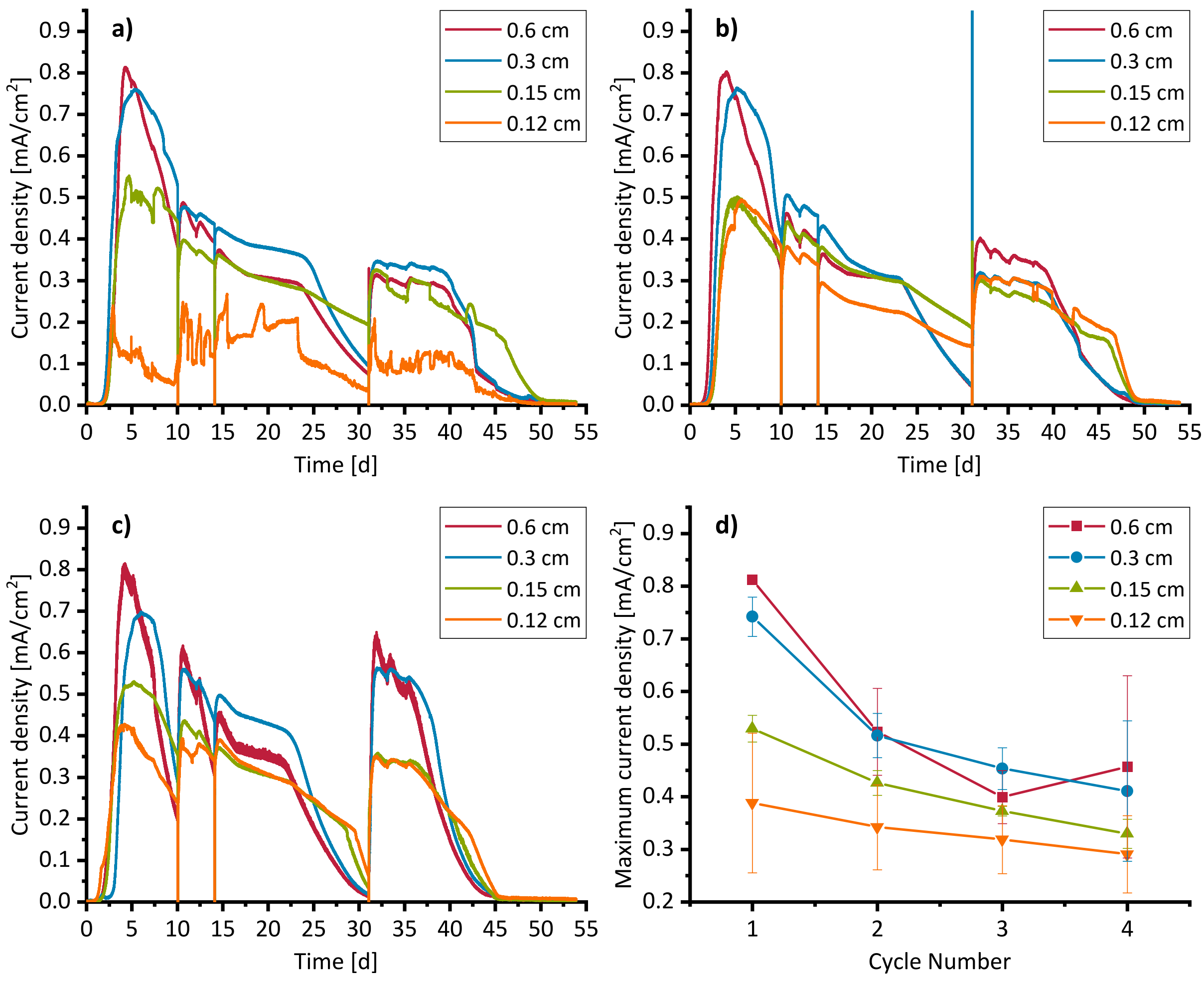

Figure S8: Chronoamperometry curves a), b), c) and maximum current densities d) of run no. 3 including the following duct diameters: 0.12 cm, 0.15 cm, 0.3 cm and 0.6 cm

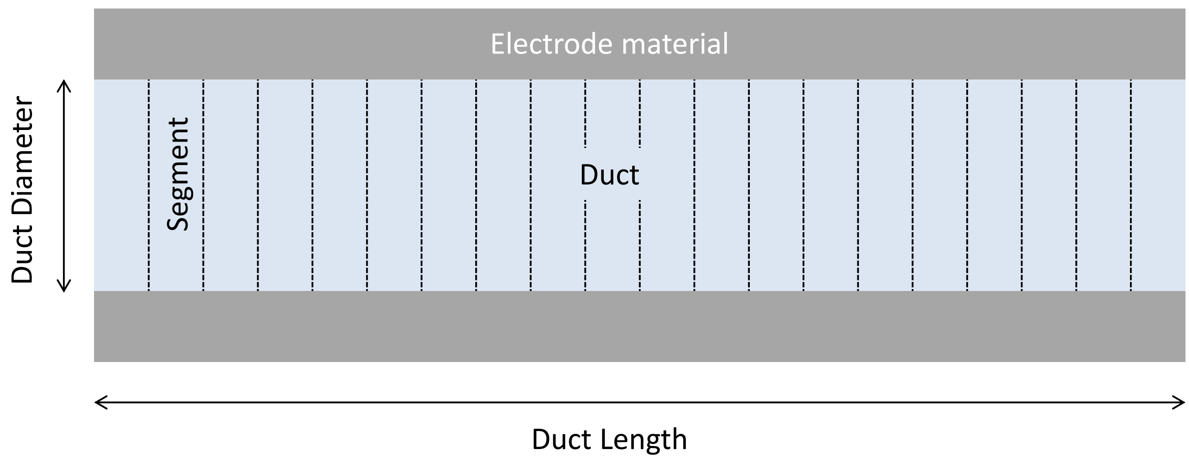

Figure S9: Schematic illustration of a duct divided into several segments for calculation of the potential-current distribution

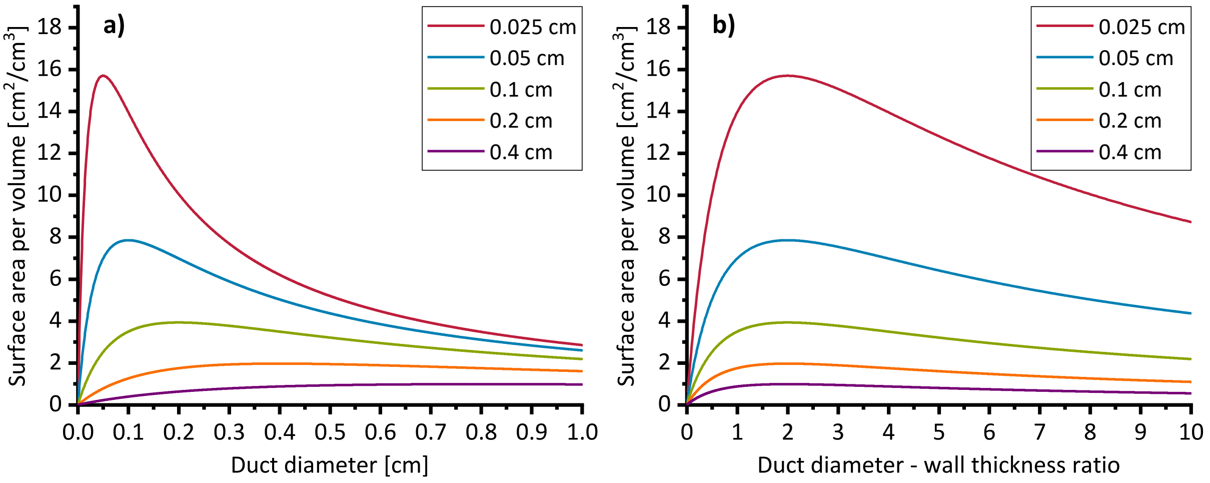

Figure S10: Calculation of surface area per volume for the model system of channels with a certain diameter d worked through a bulk material. The coloured lines refer to the wall thickness b, the diameter of the remaining material between the channels. While the surface area per volume is generally higher for lower b (figure a) the difference only be substantial for the lower diameters in figure a as a higher d reduces the number of channels and thus the density of the packing. If the system is scaled without changing its geometry (figure b) the surface area per volume decreases significantly with increasing size and the maximum surface area will always be obtained for d/b = 2.

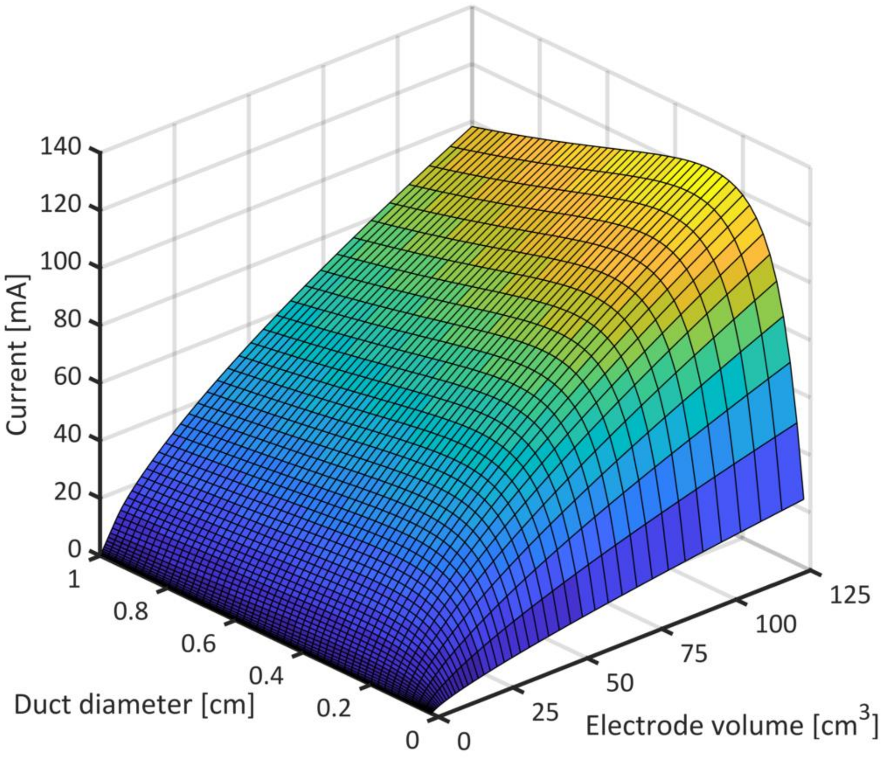

Figure S11: 3D-Plot showing the current produced plotted against electrode volume (up to 125 cm^3) and duct diameter (up to 1 cm)
